# Semantic search helper: A tool based on the use of embeddings in multi-item questionnaires as a harmonization opportunity for merging large datasets – A feasibility study

**DOI:** 10.1192/j.eurpsy.2024.1808

**Published:** 2025-01-20

**Authors:** Karl Gottfried, Karina Janson, Nathalie E. Holz, Olaf Reis, Johannes Kornhuber, Anna Eichler, Tobias Banaschewski, Frauke Nees, IMAC-Mind Consortium

**Affiliations:** 1 Institute of Applied Medical Informatics, University Hospital Center Hamburg-Eppendorf, Hamburg, Germany; 2 Department of Child and Adolescent Psychiatry and Psychotherapy, Central Institute of Mental Health, Medical Faculty Mannheim, University of Heidelberg, Mannheim, Baden-Württemberg, Germany; 3 Institute of Medical Psychology and Medical Sociology, University Medical Center Schleswig-Holstein, Kiel University, Preußerstraße 1-9, Kiel, Schleswig-Holstein, Germany; 4 German Center for Mental Health (DZPG), Partnersite Mannheim-Heidelberg-Ulm, Germany; 5 Department of Child and Adolescent Psychiatry, Neurology, Psychosomatics and Psychotherapy, Rostock University Medical Centre, Rostock, Germany; 6 Department of Psychiatry and Psychotherapy, Friedrich-Alexander-Universität Erlangen-Nürnberg (FAU), Erlangen, Germany; 7 Department of Child and Adolescent Mental Health, Friedrich-Alexander-Universität Erlangen-Nürnberg (FAU), Erlangen, Germany

**Keywords:** natural language processing, harmonization, semantic, questionnaires, big data

## Abstract

**Background:**

Recent advances in natural language processing (NLP), particularly in language processing methods, have opened new avenues in semantic data analysis. A promising application of NLP is data harmonization in questionnaire-based cohort studies, where it can be used as an additional method, specifically when only different instruments are available for one construct as well as for the evaluation of potentially new construct-constellations. The present article therefore explores embedding models’ potential to detect opportunities for semantic harmonization.

**Methods:**

Using models like SBERT and OpenAI’s ADA, we developed a prototype application (“Semantic Search Helper”) to facilitate the harmonization process of detecting semantically similar items within extensive health-related datasets. The approach’s feasibility and applicability were evaluated through a use case analysis involving data from four large cohort studies with heterogeneous data obtained with a different set of instruments for common constructs.

**Results:**

With the prototype, we effectively identified potential harmonization pairs, which significantly reduced manual evaluation efforts. Expert ratings of semantic similarity candidates showed high agreement with model-generated pairs, confirming the validity of our approach.

**Conclusions:**

This study demonstrates the potential of embeddings in matching semantic similarity as a promising add-on tool to assist harmonization processes of multiplex data sets and instruments but with similar content, within and across studies.

## Introduction

In large epidemiological and clinical studies in the field of mental health, data not only span a wide array of constructs, including behavior, cognitive patterns, personal attitudes, or beliefs that primarily come from questionnaires but also a wide array of instruments used to capture these constructs, both across and within studies and cohorts [1,2]. If researchers aim to merge data from two or more studies for analysis, the gold standard is to use data collected with identical questionnaires or, at the very least, with comparable (sub)scales of these questionnaires, thereby ensuring adherence to standardized values. This can be achieved through the recourse to catalogs that provide an overview of measures used in different cohorts (e.g., https://lifecourse.melbournechildrens.com/cohorts/; https://www.cataloguementalhealth.ac.uk). However, the aspect of comparability already raises questions as it can be assessed and treated in different ways. Moreover, given the large number of existing studies and cohorts in mental health [3–6], it is often evident that studies or cohorts of interest have not used consistent questionnaires to measure the same constructs. Additionally, in longitudinal studies or cohorts, different questionnaires are sometimes used at various assessment points over time.

Therefore, it is particularly important to develop methods that enable the utilization of this data across different studies and projects for population-based analyses [7]. In this respect, there is a growing emphasis on expanding individual datasets through ex-post harmonization, which involves merging data to create a unified, comprehensive, and semantically fitting dataset that preserves the validity and reliability of the outcome measures [8–12]. This involves standardizing the available questionnaire data down to the item level [6,13–15]. It requires a comprehensive understanding of the significance of each collected variable and methodological consistency across studies, including the definitions, measurement instruments, and data collection protocols used. By thoroughly examining these aspects, researchers can identify comparable variables, mitigate potential biases, and enhance the reliability and validity of harmonized datasets, ultimately facilitating robust cross-cohort analyses and more accurate scientific inferences. When data on common constructs are available only from different questionnaires, this harmonization extends beyond variables to individual questionnaire items to ensure that the items from different questionnaires measure the same and thus can be treated as indicators of the same construct. In this respect, it is important to determine the similarity of items from the questionnaires, which can be done by experts from the field, and thus on a subjective level, or by natural language processing (NLP)-based algorithms. For example, questionnaires like the Alcohol Use Disorders Identification Test (AUDIT) [15] and the Michigan Alcohol Screening Test (MAST) [16] have similar questions about alcohol consumption. For example, AUDIT’s question 8 (“How often during the last year have you been unable to remember what happened the night before because you had been drinking?”) inquiries about the frequency of memory lapses due to alcohol intake over a specific period. Similar content is assessed in MAST question 2 (“Have you ever awakened the morning after drinking the night before and found that you could not remember a part of the evening?”), albeit with different wording and structure. In this case, the question texts provide relevant information for assessing the potential for harmonization between the questionnaires. Thus, the question text serves as a form of meta-data description that can be beneficial for identifying semantic similarities across questionnaires [17]. This semantic harmonization is a pre-requisite for any further harmonization processes, such as syntactical harmonization. Syntactical harmonization involves addressing distinct data types and the data granularity of the responses (e.g., AUDIT – numeric variable versus MAST dichotomous variable). This syntactical alignment can only be performed after semantic matching has been established. Finally, it is essential to implement a manual integration step, that captures afore mentioned aspects regarding existing catalogs and frameworks as well as the expert rating(s). This also includes technical interoperability tools such as meta-data repositories and uniform models as well as code systems, terminologies, and classifications that align the data harmonization with the FAIR principles [18]. Examples such as LOINC, SNOMED CT, RxNorm, or ICD-10 play a significant role in aligning data and are therefore increasingly used for this task [19-22]. These tools are specifically designed for incorporating lists and standardized information, and the related information should be captured also in the context of NLP-based semantic similarity tests. To facilitate the integration of a manual as well as a user-/expert-based perspective also means the use of advanced features to support various display options, resolutions, and value ranges. This integration requires advanced features to support various display options, resolutions, and value ranges. Beyond simply considering the degree of matching or network analysis, this enables subjective evaluation levels, important especially for critical elements or even already those that are set in a relatively high similarity range of 60%–70%, which still raises the question of the appropriateness of the data merging step. This approach could also initiate further processes that lay the groundwork for future studies on item combinations and their assessment, contributing to a more in-depth evaluation. The consideration of merging and comparing instruments in different languages is finally another important feature because often cohorts from different countries are available and can bring corresponding benefits, but standard instruments are not always validated for different languages.

In summary, comparing data from large and complex datasets in terms of constructs, significance, and indication levels is an immense and often unmanageable task, especially regarding the comparability of questionnaire data from different instruments. Given that manual harmonization in this respect is error-prone and resource-intensive [9,10,12,16], the full utilization of available multi-cohort data and missed potential opportunities is hindered [23,24]. In this respect, automated methods that can pre-select semantic item pairs based on criteria such as formulation and content using NLP, such as transformer-based embeddings [25,26], may offer a solution. Embedding techniques from advanced language models convert words or sentences into numerical representations, allowing computers to process and compare them. These embeddings represent data in a way that preserves meaning, facilitating the identification of semantically similar items. This offers significant advantages over previously prevalent frequency-based approaches such as the frequency-inverse document frequency (TF-IDF) method [27–29]. Results are represented as distances in a virtual space, where words or sentences with similar meanings are closer together, allowing for a measurable comparison of their similarity. These techniques have shown high performance in tasks involving text-based semantic similarity comparison tasks [30–32], rendering them appropriate for semantic search within the framework of data harmonization [33]. Platforms such as Hugging Face [34], spaCy [35], or services like ChatGPT [36,37] enable these techniques. In the present article, we employ automated methods, together with advanced and extensive representation and visualization features, and language-based testings, to detect semantically similar questionnaire items related to mental health, while still making use of available terminology standards such as LOINC and integrating the user perspective. This is based on the aim to target some of the challenges when pooling cohort data, including too imprecise estimates and misalignment between cohorts [23]. We evaluate this approach with a prototype application, called “Semantic Search Helper,” which is seen to assist harmonization processes with data from different studies, cohorts and based on multiplex data sets, and thus to “help” the researchers and experts in their decision on whether or not to merge heterogeneous data obtained with different instruments. We present a use case analysis with data from various large-scale health-related cohort studies and expert ratings for further validation.

## Methods

### Health-related cohort studies and related questionnaire data

For the use case analysis, we used questionnaire data from four heterogeneous cohorts (see Table 1) merged in the context of the IMAC-Mind (Improving Mental Health and Reducing Addiction in Childhood and Adolescence through Mindfulness: Mechanisms, Prevention, and Treatment) consortium [38]: ROLS (Rostocker Längsschnittstudie) [39]; MARS (Mannheim Study of Children at Risk) [40–42]; FRANCES (Franconian Cognition and Emotion Studies); [43] and POSEIDON (Pre-, Peri-, and Postnatal Stress: Epigenetic Impact on Depression). Within IMAC-Mind, these cohorts have been selected with the aim to increase knowledge about the development of addiction during childhood and adolescence enlarging the population size, enhancing the statistical power of evidence, and thereby magnifying the study’s impact and validity. The cohorts consist of 1329 participants encompassing 44 instruments (see Table 1). For demonstration purposes, we utilized a subset of 31 licensed instruments with a total of 1458 item texts (see Table 2). These selected instruments and items cover a diverse array of concepts and constructs with varying syntactical structures. The inclusion of both German and English items allows us to investigate the potential for multilingual harmonization.

**Figure 1.**
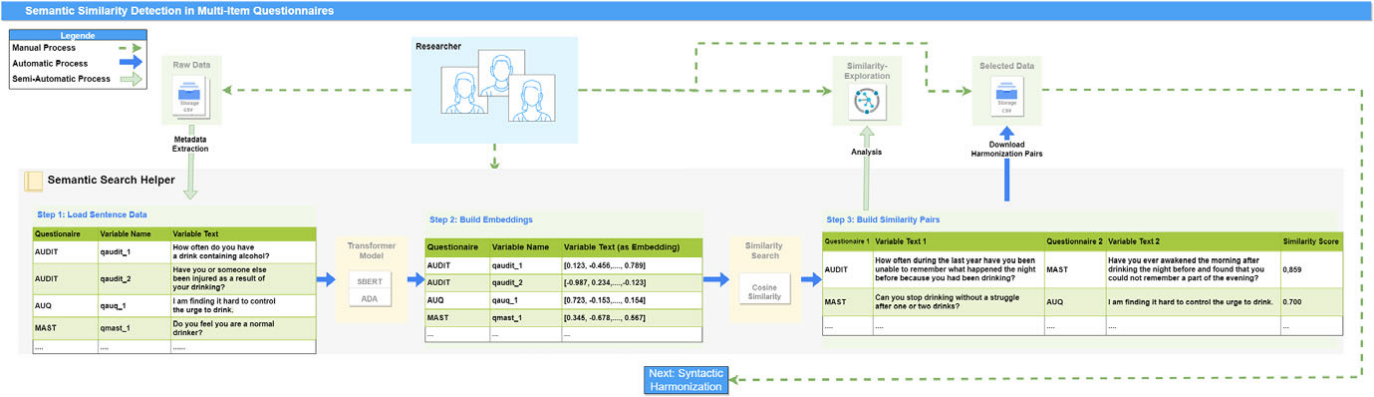
Workflow concept for detecting semantic similarities within multi-item questionnaires. The process involves: (1) inputting sentence data, including variable names and text; (2) converting text into vectors using models like SBERT or ADA; (3) building similarity pairs to identify semantic matches and generating scores; (4) selecting and downloading pairs for further analysis. Color coding indicates manual, automatic, and semi-automatic processes.

**Table 1.** Overview of IMAC-mind cohort studies

Study name	Description	Subject Count	Instrument count
FRANCES	Prospective cohort study aimed to explore the effects of prenatal risk factors on cognitive and emotional development with an additional focus on biomarkers. The data were observed by mothers and their children in early school age.	248	17
MARS	Epidemiological study following the long-term outcome of early risk factors including basic cognitive, motor, and non-cognitive abilities and social as well as academic achievement. Data were obtained in 10 waves, following participants at risk for later developmental disorders from birth to 25 years.	384	12
POSEIDON	Prospective cohort study contained a sample of mothers and their infants regarding pre-, peri- and postnatal stress in human and non-human offspring. The design of the study included four waves, where the children’s data was observed from the prenatal period till the age of four.	401	7
ROLS	Epidemiological study with the main goal focused on clarification of interactions of perinatal and environmental risk factors influencing personality development including mental health and problem behavior. The sample includes seven assessment waves. Here, participants were 0–36 years old.	296	8
Total		1329	44

**Table 2.** Overview of questionnaires used in the studies

Questionnaire (abbreviation)	Studies used	Items count
ADHS-Langzeit-Erfassung (ADHS-LE) (51)	Frances	64
Alcohol dependence scale (ADS) (52)	Mars	20
Adaptives Intelligenz Diagnostikum (AID) (53)	Frances	20
Alcohol consumption interview		7
Advanced personality questionnaire, second version (APQ-2) (54)	Frances	72
Alcohol use disorders identification test (AUDIT) (55)	Mars	10
Beck depression inventory (BDI) (56)	MARS	22
Belgian violent behavior assessment (BelVa)	Frances	23
Comprehensive behavioral questionnaire (CBQ) (57)	Frances	94
Eltern-belastungs-screening (EBSK) (58)	Frances	63
Edinburgh postnatal depression scale (EPDS) (59)	Frances, Poseidon	10
Environmental stress index (ESI) (60)	Frances	20
ADHD-specific behavior assessment form (FBB-ADHS) (61)	Frances	20
Anxiety-specific behavior assessment form (FBB-ANZ) (61)	Frances	33
Depression specific behavior assessment form (FBB-DES) (62)	Frances	28
Social behavior assessment form (FBB-SSV) (62)	Frances	25
Fagerstrom test for nicotine dependence (FTND) (63)	Mars	7
Inventory of depressive symptomatology (IDS) (64)	Frances, Mars	24
Kindergarten adjustment scale (KiGa)	Rols	30
KINDL questionnaire for measuring health-related quality of life in children and adolescents (KINDL) (65)	Frances, Mars	24
Life experiences survey (LES) (66)	Poseidon	54
NEO personality inventory, long version (NEO-FFI long version) (67)	Rols	241
NEO personality inventory, short version (NEO-FFI short version) (67))	Mars, Poseidon	30
Perceived stress scale (PSS)	Rols	133
Strengths and difficulties questionnaire (SDQ) (68)	Frances	25
Smoking habit interview	Frances, Mars, Poseidon, Rols	9
Social support questionnaire (69)	Rols	69
State-trait anxiety inventory – short form (STAI-S) (70)	Mars, Poseidon	20
State-trait anxiety inventory – trait form (STAI-T) (70)	MARS, Poseidon	20
Stress coping inventory (SVF) (71)	Rols	112
Typical personality factors questionnaire (TPF) (72)	Rols	129
Total Items		1458

### The process of semantic similarity search in the context of merging health-related datasets and the implementation of respective features into an online tool called “Semantic Search Helper”

The Maelstrom Institute has established protocols for harmonizing health-related datasets [44]. Acknowledging that the guidelines do not extensively cover the semantic search process, our initial emphasis was on outlining the pertinent steps of this process from a researcher’s perspective. To aid researchers in manually identifying semantic harmonization opportunities in multi-item questionnaires and studies, we have delineated our approach into the following steps:

### The manual search process

Firstly, we implemented a manual search process from the perspective of scientists defining the following activities as core features:

1. *Collection of questionnaires and their items:* Researchers need to be able to easily check relevant studies and their questionnaires for semantic similarities. Established sources such as LOINC (Logical Observation Identifiers Names and Codes) [45] and MDM (Medical Data Models) [46] as well as own data sources are utilized to efficiently identify harmonization opportunities. Moreover, the aforementioned available catalogs also provide a valid source of information that can be integrated into the “Semantic Search Helper” toolbox.
2. *Identification of similar questionnaire content:* Questionnaires and their items are compared in terms of concepts and semantic similarity to form groups of similar questionnaires.
3. *Filtering relevant semantic pairs:* Semantically relevant pairs are selected based on specific research questions, allowing for efficient filtering through large volumes of data.
4. *Preparation for syntactic harmonization*: The selected item pairs are scrutinized for syntactic consolidatability to assess the granularity of the questions and ascertain their harmonization potential.

### Identification of methods and techniques to support semantic similarity search

To improve the efficiency of the manual process, we explored applicable technological solutions. We focused on using advanced language-processing techniques, especially methods that help identify meaning in sentences (particularly attention-based embeddings). Sentence Embedding models such as SBERT and OpenAI embeddings were evaluated for their potential to facilitate semantic comparisons more effectively and efficiently than traditional nontransformer-based methods (similar to [17]).

The platform Hugging Face offers many open-source models that provide a text/sentence-to-vector/embedding interface [34]. Our model selection was based on two characteristics: (a) the model was already tested for text similarity search tasks, and (b) the model was capable of handling multiple languages.

We selected the open-source sentence transformer model SBERT [47] and the GPT-3 model ADA (“text-embedding-ada-002”) for our use case. The models have the following characteristics:

#### OpenAI embeddings

The OpenAI API provides an easy-to-use framework based on large datasets for converting texts into embeddings. We utilized the “textembedding-ada-002” model accessible via OpenAI’s REST API endpoint. We used the proposed configuration with the “cl100k base” embedding encoding. The questionnaire text was used as input, and we called the OpenAI Embedding API with the provided Python script (Python 3.8.) to create an embedding for each questionnaire.

#### SBERT sentence embedding

Sentence embeddings are a further option to make embeddings out of texts. This type of embedding is a variant of the renowned encoder BERT (Bidirectional Encoder Representations from Transformers), widely utilized in various applications [32, 49]. For our use case, we employed SBERT, a model optimized for sentence embeddings [48, 50]. We utilized the openly available model “sentence-transformers/paraphrase-multilingual-MiniLM-L12-v2,” capable of handling multiple languages. We adhered to the guidelines for setting up text-to-embedding translation.

#### Embedding-related algorithms

Clustering is a well-known method in the realm of embeddings. K-nearest neighbors can be relatively easily calculated based on given vectors and distance metrics. The visualization of high-dimensional vectors can be achieved by dimension reduction techniques such as Uniform Manifold Approximation and Projection (UMAP) [50], which simplifies complex structures into understandable formats, preserving the essential characteristics of the data. Discovering topics through embeddings represents another algorithm linked to embeddings, potentially valuable for data harmonization, especially with unfamiliar datasets. BERTopic employs a neural approach to topic modeling, utilizing a class-based TFIDF mechanism [51]. Additionally, we selected the open-source library Faiss [52] for efficient similarity search.

#### Data visualization methods

To enhance understanding of the underlying data, data visualization techniques are crucial. Advanced visualization tools such as Plotly [53], ECharts [54], and Seaborn provide valuable means to illustrate relationships among elements and highlight critical details. These selected tools facilitate the creation of dynamic plots and charts that are interactive and user-friendly, making complex data more accessible. Another advantageous visualization technique we found useful in the realm of data harmonization is the utilization of interactive networks [55], which can be especially effective during the search and filtering phases. Interactive networks empower users to dynamically explore connections and patterns within the data, providing a potent means to visualize relationships and dependencies.

### Pre-testing of transformer-based embeddings in the context of data harmonization with a group of domain experts

We conducted a pre-test with seven experts to evaluate the use of transformer-based embeddings in data harmonization, focusing on the feasibility of employing cosine similarity derived from these models. Domain experts from the authors’ network with prior experience in the field of psychology and cognitive neuroscience and in working with questionnaire data were contacted and invited to participate in the pretesting. In total, 7 experts rated 20 candidates for semantic similarity, assessing each for suitability for harmonization, similarity score, and providing qualitative feedback. We also provided an open comment opportunity to capture additional information on the evaluation procedures.

The selection of the questions was carried out as follows: For each of the 31 instruments, three groups were built based on the cosine score between the pairs of questions. These were the five most similar pairs, the five pairs closest to the overall cosine mean, and the five least similar pairs. For every instrument, one of the groups was randomly chosen, leading to 155 pre-selected pairs. We then randomly selected 20 pairs from the pre-selected ones to ensure that each instrument had the same chance to be included and that the three similarity groups were represented in the final selection.

We analyzed responses to categorize semantic pairings as positive or negative, aiding in the determination of their suitability for harmonization.

### Prototype development based on the identified workflow

Based on the results from the pre-testing of embeddings with a small group of users, we developed a software prototype using Python 3.8 [56] and the Streamlit library [57]. This prototype will incorporate algorithms and methods mentioned in the previous sections, such as UMAP for dimension reduction, Faiss for efficient similarity search, and SBERT along with ADA for embedding generation. The prototype demonstrates the feasibility of a supported harmonization workflow using the IMAC-Mind dataset as a use case.

## Results

### Workflow development for supported semantic search tasks

Based on the analysis of the manual process and the algorithms identified appropriately during the technical assessment, a concept for supported semantic harmonization and its implementation was developed (see Figure 1). The workflow incorporates the main activities of researchers in identifying harmonization candidates and includes the algorithms and methods described in the Methods chapter. The concept necessitates the sequential processing of the questionnaires, ultimately resulting in a potential list of harmonization pairs, which can be used for further syntactic verification (not covered by this concept). This concept resulted in four main steps as a workflow of a semantic search task:

1. *Providing questionnaire data:* The ability to upload questionnaires and their corresponding items is essential to initiate the automation process. Sources such as the Logical Observation Identifier Names and Codes (LOINC) database [45], or the Portal of Medical Data Models (MDM) [46] can provide well-structured questionnaire metadata. Uploading Excel (.xlsx) or CSV (.csv) files is advantageous. For item matching, at minimum, the name of the questionnaire and the text of each question are required. Additional information such as variable names or study names may be helpful for the harmonization evaluation process.
2. *Using models to represent sentences:* Generate a vector for each sentence using advanced NLP models like ADA or a comparable model. These vectors represent the semantic meaning of each sentence.
3. *Cosine similarity calculation*: Calculate the cosine similarity between the vectors of all sentences from the questionnaires. Pair each sentence with every other sentence to determine similarities.
4. *Filtering the list of pairs:* Filter the list of sentence pairs based on relevant characteristics to identify the best matching pairs. These characteristics include the strength of similarity or specific instruments.

**Figure 2.**
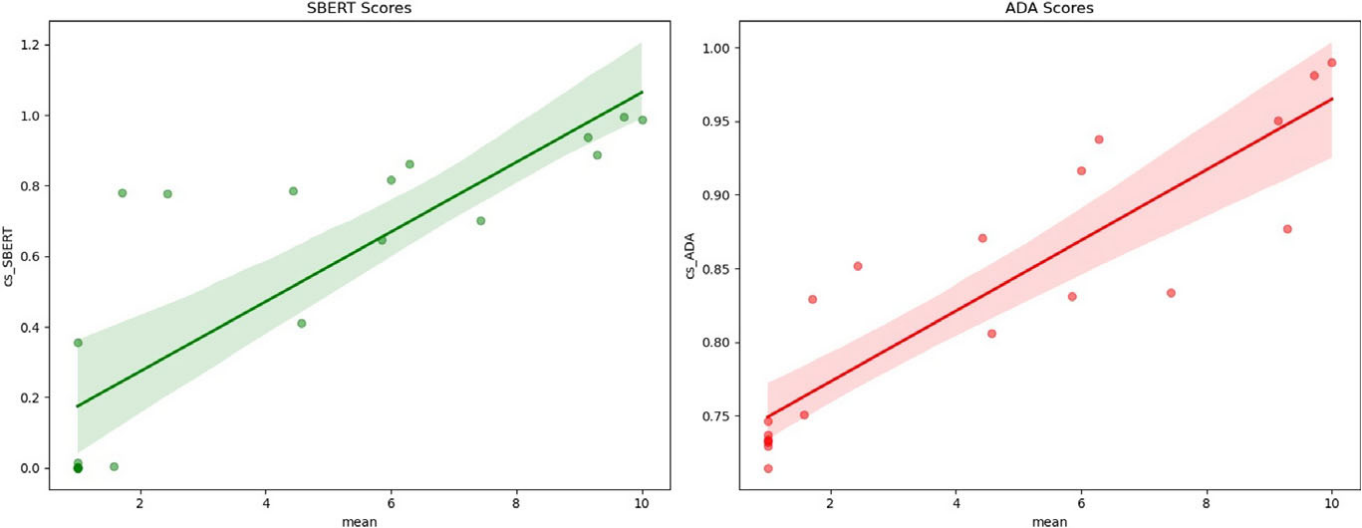
Mean score responses and model-based similarity scores. The correlation between average score responses (*x*-axis) and similarity scores generated by the embedding models (*y*-axis). Scatter plots: score responses versus model similarity scores. Each plot represents the correlation between mean score responses and similarity scores for SBERT (green) and ADA (red) models. The overall trend in the relationship between evaluative scores and model-derived similarity metrics is indicated by linear regression lines.

### Evaluation within the scientific area: pre-testing results of expert ratings

The group of participants (*N* = 7) had a high level of experience in the field of data harmonization, averaging 7.14 years (standard deviation [SD] 6.440), working both in the clinical (43%) and research (57%) fields (psychological and neuroscientific research). The relevance of the topic, data harmonization, was rated with a mean of 8.14 (SD: 1.069) on a 10-point Likert scale, indicating high relevance. Table 3 shows the 20 examples that were used for the pre-testing with the corresponding similarity scores from the SBERT and ADA Models.

**Table 3.** Comparison of semantic similarity scores for questionnaire item pairs using SBERT and ADA algorithms

Questionnaire item pairs	Cosine score SBERT	Cosine score ADA	Rating “yes” count	Rating “no” count
“I have trouble sleeping at night” (ADS) – “Religious ideation” (CAPE-42)	0.000	0.729	0	7
“Alcohol makes people feel like they can” (AEQ) – “I am feeling an emotional need for alcohol.” (AUQ)	0.781	0.829	0	7
“Feeling irritable or easily angered.” (ANSQ) – “Leicht gereizt oder genervt fühlen.” (BSI)	0.887	0.877	7	0
“I would like to drink right now.” (AUQ) – “Feeling worthless or inferior.” (BSI)	0.014	0.733	0	7
“Continued alcohol use despite having persistent or recurrent social or interpersonal problems caused or exacerbated by the effects of alcohol.” (BASIC) – “I say things without thinking.” (BIS-11)	0.001	0.732	1	6
“I am impatient” (STAI-T) – “Feeling tense or keyed up.”(BSI)	0.410	0.806	4	3
“Feeling that others are against you” (BSI) – “I am careful and thorough in my work.” (NEO-FFI)	0.000	0.715	0	7
”Have people Annoyed you by criticizing your drinking?” (CAGE) -” Feeling lonely.” (BSI)	0.005	0.751	0	7
“When I’m faced with a stressful situation, I make myself think about it in a way that helps me stay calm” (ERQ) “Change in church activities” (SRRS)	0.000	0.737	0	7
“How often do you smoke cigarettes at present?” (ESPAD) – “How soon after you wake up do you smoke your first cigarette?” (CDSS)	0.786	0.871	2	5
“How many cigarettes do you smoke per day?” (FTND) – “How many cigarettes a day do you smoke?” (CDSS)	0.994	0.981	7	0
“Becoming easily annoyed or irritable” (GAD-7) – “Feeling easily annoyed or irritated.” (BSI)	0.937	0.950	7	0
“Feeling trapped or caught” (HSCL-25) – “Feeling trapped or caught.” (BSI)	0.986	0.990	7	0
“Alcohol-related problems in past year” (LDH) – “Drinking alcohol helps people deal with life’s problems” (AEQ)	0.778	0.852	1	6
“Can you stop drinking without a struggle after one or two drinks?” (MAST) – “I am finding it hard to control the urge to drink.” (AUQ)	0.701	0.834	5	2
“Poor appetite or overeating” (PHQ) – “ Loss of appetite” (CSI)	0.816	0.916	5	1
“Important activities given up or reduced” (SCID-SUD) – “Putting less effort into things” (AES)	0.647	0.831	6	1
“Please mark on this ladder the rung where you think you stand, relative to other people in the United States, in terms of education, money, and jobs.” (SSS) – “I am interested in other people’s problems.” (NEO-FFI)	0.355	0.734	0	7
“I feel good” (STAI-C) – “Drinking too much alcohol can be dangerous or fatal” (AEQ)	0.001	0.746	0	7
“I feel nervous and restless” (STAI-S) – “I feel more nervous and anxious than usual” (ADS)	0.861	0.938	6	1

#### Similarity candidate ratings

Candidate pairs were rated by the experts according to the models: Within the group of positive candidate pairs, the pairs exhibited a mean agreement by the experts to be classified as positive of 87.0% (95% CI, 75.4–98.7), while the negative candidate pairs showed a mean agreement of 94.8% (95% CI, 88.3–101.2).

#### Similarity score rating

Similar findings were also observed for the 10-point similarity rating scale. Moreover, the sentence embedding (SE) method is strongly related to human ratings (0.837, *p* < 0.001), see Table 4 and Figure 2.

**Figure 3.**
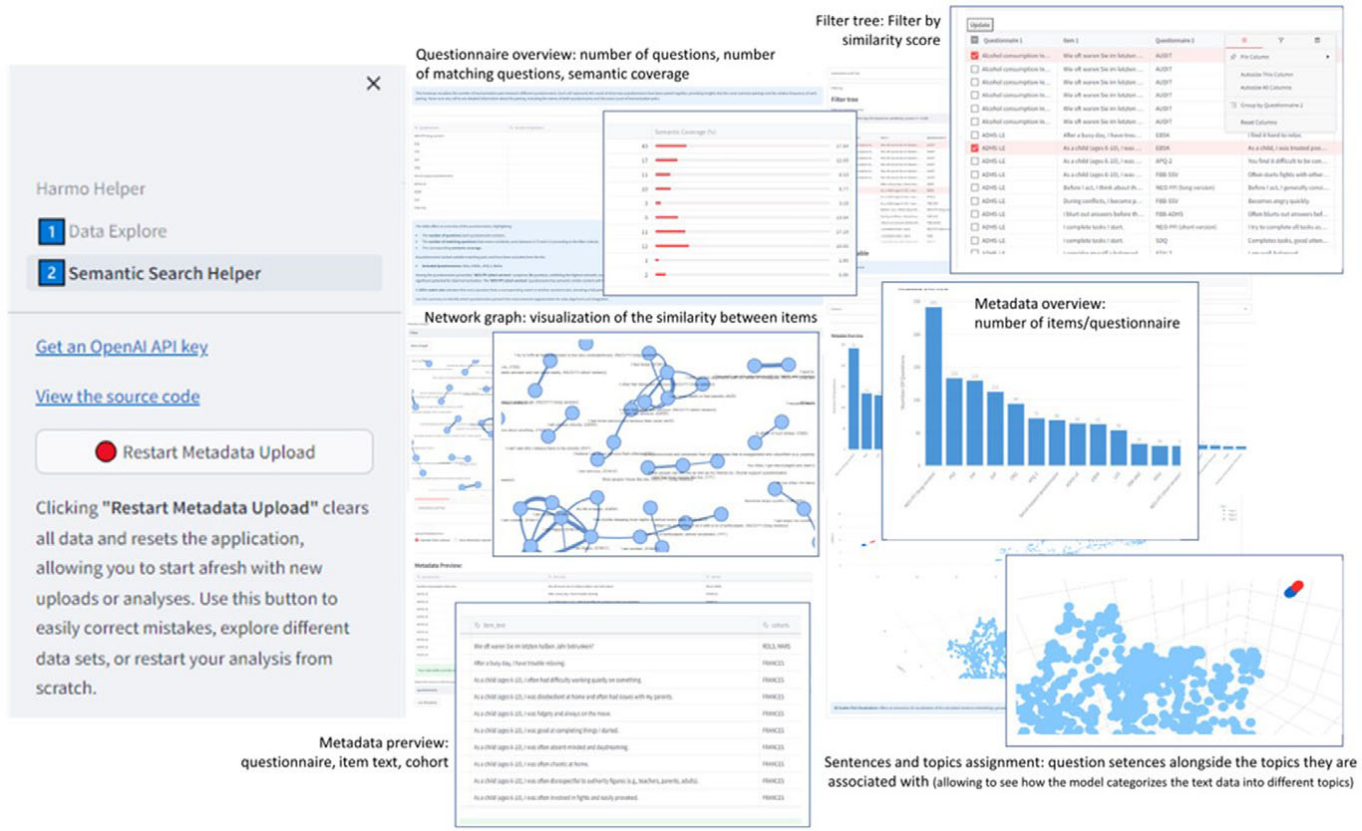
‘Semantic Search Helper’ application interface. The user interface (UI) with different stages of the harmonization process: A bar chart overview of metadata distribution and a scatter plot for visualizing data points in semantic dimensions (Bottom Section). A filter tree for selecting specific data items and a filtered table displaying data based on applied filters (Right Section). Table with survey questions and semantic similarity scores, and a bar chart showing semantic coverage percentages (Top Section). A network graph visualizing semantic connections between questionnaire items (Middle Section). This interface facilitates the comparison of semantic similarities across survey questions, streamlining the data harmonization workflow for researchers.

**Table 4.** Spearman correlations and 95% confidence intervals

Comparison	Correlation coefficient	95% CI lower bound	95% CI upper bound
Ratings versus SBERT	0.907	0.751	0.962
Ratings versus ADA	0.928	0.808	0.974

Analysis of the central tendency and variance shows that 11 pairs have a low variance (SD <1) highlighting pairs with a semantically clear rating. Especially for these pairs, the correlation between PSS and SES was 0.974 (*p* < 0.0001) and perfectly correlated, indicating that the SES highlights clear semantic similarity in the high score ranges (≥0.88) and clear semantic dissimilarities in the low score ranges (≤0.35). The remaining nine pairs had a greater variance for the PSS indicating that the pairs were harder to rate. The pairs (ID: 720) (LDH) “Alcohol-related problems in the past year” – (AEQ) “Drinking alcohol helps people deal with life’s problems” and the pair (ID: 385)” (STAI-T) “I am impatient” – (BSI) “Feeling tense or keyed up.” had the highest variability (SD of 2.936 and 2.760).

We interpreted the pre-testing results as positive and continued our work with a prototype implementation to support the search for harmonization opportunities with transformer-based embeddings.

### Prototype development: Workflow implementation and application to the IMAC-Mind dataset

Our implementation of the workflow concept resulted in a prototype application called “Semantic Search Helper” (see Figure 3). We tested automation functionalities with the list of unlicensed instruments in Table 2.

**Figure 4.**
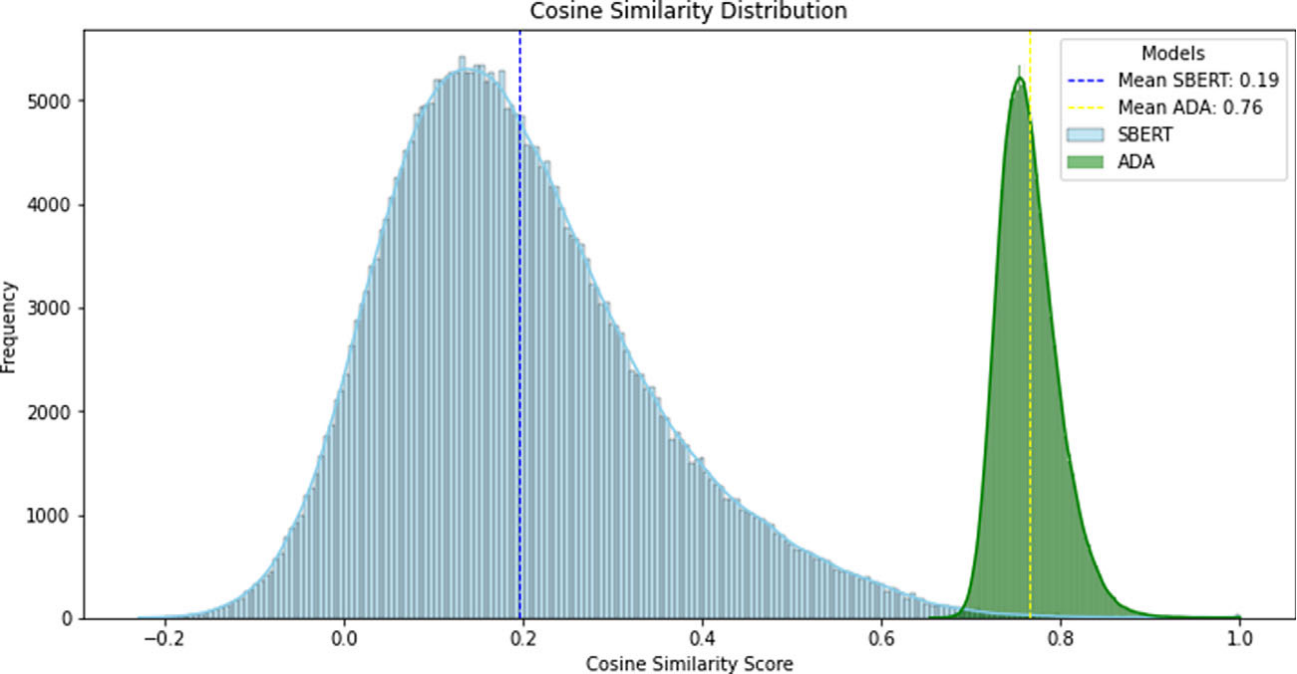
Cosine similarity distributions for SBERT (light blue) and ADA (green). SBERT has a mean of 0.19 (blue line) and ADA has a mean of 0.76 (yellow line).

#### Embeddings and cosine similarity

The prototype application created 994.609 potential pairs in total and 15.085 with Faiss out of the IMAC-Mind dataset with 1458 questions and 31 instruments. The distribution of cosine similarity scores for the two models is shown in Figure 4. SBERT has a mean cosine similarity score of 0.195 (SD: 0.149), while ADA has a mean cosine similarity score of 0.765 (SD: 0.033), showing that the value scores are different between the models.

In the following, we describe only the results derived from the SBERT model, as there were no significant differences between SBERT and ADA.

#### Clustering and topic building

The clustering and BERTopic-based topic building revealed between 3 and 142 topics or clusters for the 1458 sentences. A clear clustering was observed with alcohol-related questions (Topic 1: *N* = 21) and smoking-related questions (Topic 2: *N* = 16). The remaining questions (Topic 0: *N* = 1421) were clustered together and presented a semantically unclear view (in Supplementary Table S1).

#### Relevant pair filtering

For our analysis, we selected all pairs with a similarity score higher than 0.751, which was the lower confidence interval (CI) bound obtained from the pre-testing. This selection resulted in 312 pairs (in Supplementary Table S2).

Based on these 312 pairs, the content coverage of the questionnaires in the application was displayed using a dependency wheel visualization and a table. The NEO (long version) with 62 items, which showed semantic similarities with other questionnaire items (cosine similarity >0.751), had the highest number of matches. With a total of 241 items, this accounted for a relative share of 25.73% of the questionnaire items covered. The NEO (short version) showed a relative coverage of 100%, covering all 30 questions, thus having the highest value among all 31 questionnaires, followed by STAIS-S at 85% and STAIS-T at 70% (in Supplementary Table S3). The “NEO-FFI (short version)” questionnaire has semantic similar content with the following eight questionnaires: (EBSK, SVF, TPF, STAI-S, STAI-T, NEO-FFI (long version), ADS, ADHS-LE). In general, two questionnaires (KINDL, BelVa) did not have content matches based on the chosen similarity score. This information is presented to the user both visually and in a summary of the interface.

#### Quantifying the automation potential

By considering the described findings and the strong correlations observed between the SBERT, ADA models, and human raters, we developed a theoretical hypothesis regarding the potential benefits of this methodology across the involved cohorts. Assuming that the confidence intervals for positive and negative candidate pairs can categorize the entire pool of harmonization candidates, the following results were obtained based on the estimated percentage of data that embedding models can automatically process without human intervention.

For our use case, 0.35% (*N* = 1,132) of the pairs are classified as positive candidate pairs based on a confidence interval (CI) of 0.676 to 0.912, while 73.39% (*N* = 235,826) are classified as negative candidate pairs based on a CI of 0.737–0.800. This indicates that 73.74% (*N* = 236,958) of the data can be automatically processed using embeddings, whereas 26.26% (*N* = 83,041) is unclear to the SBERT model and requires human review. The findings are comparable to the ADA model, with only 0.83% (*N* = 2,665) of the pairs being within the CI (0.862 to 0.945) for positive candidate pairs and 66.55% (*N* = 213,845) of the pairs being within the CI for negative candidate pairs. Consequently, 67.38% (*N* = 216,510) of the candidate pairs can be automatically processed with the ADA embeddings. Exactly 32.62% (*N* = 103,489) of the candidates remain uncertain and require human verification. This initial analysis suggests that, based on the confidence intervals applicable to the original dataset, a minority of possible candidates (26% and 32%) would require human verification for the use case considered.

## Discussion

Our work shows that using NLP techniques to harmonize questionnaire data can be significantly beneficial. By employing semantic search methods, we compared 1458 items from 31 questionnaires. Similar studies [6,9,12,15] indicate that harmonization is time-consuming, resource-intensive, and rarely scalable. Our prototype streamlines the search process, assessing harmonization potential and providing preliminary insights, both considering standardized sources of information including terminologies, the language aspect, and providing researcher-friendly and usable presentation layers, before actual data integration. This approach quantifies insights that would otherwise only be roughly estimated.

This approach can enhance mental health research by integrating and comparing data from diverse populations, improving statistical power, and identifying subtle health trends and associations. For example, studying risk factors for alcohol use disorders (AUDs), can address the challenges posed by differing data collection methods and variable definitions across studies. Researchers can use the “Semantic Search Helper” to for example investigate the combined effects of genetic predispositions, early-life experiences, and socioeconomic status on AUD development across different demographic groups and also if information and data stem from a diverse set of instruments. This improves statistical power to detect associations between risk factors for AUD and outcomes, even between subgroups with smaller sample sizes, and can enhance generalizability and facilitate the identification of commonalities and differences in AUD risk factors. Harmonized data enables the exploration of interactions between various risk factors and the identification of potential mediators (e.g., mental health conditions) not only in AUD development but also in other mental health areas, where “Semantic Search Helper” can be applied in a similar fashion. It also supports meta-analyses and replication studies, thereby strengthening research findings. Our prototype can process 67.38 to 73.74% of potential candidates without human intervention, significantly reducing manual efforts and conserving resources. Expert testing during the conceptualization phase shows high agreement between semantically equivalent and expert-considered harmonizable items, proving the validity and usefulness of semantic embeddings for data harmonization of large health-related cohorts. It is important to note that, as the name suggests, the “Semantic Search Helper” serves as a tool to support the harmonization process by quantifying the comparability of questionnaires, thus facilitating their potential integration for specific research questions and analyses. This tool tests the semantic similarity of individual items while leveraging available standard terminologies, considering language-based aspects, and involving researchers and experts in the search process. It achieves this, in part, through various visualization and summary features that provide researchers with an intuitive sense of the data, which is valuable not only in cases where similarity is marginal but also to still consider deep clinical expertise and in the end transparency of the whole process. While our results are promising in identifying opportunities for semantic harmonization, it is further important to acknowledge the challenges that this emerging technology presents. Although NLP techniques can efficiently analyze semantic content, there is still a risk of over-reliance on automated methods at the expense of thorough documentation and description of the measurement instruments used. Such a development could contravene the principles of FAIR and impact the long-term traceability of collected data. It is therefore essential to employ NLP as an additional tool for the manual recording and documentation of meta-data, rather than as a replacement for it. Future studies should further compare semantic similarity and harmonization possibilities, consider multilingual questionnaires, and utilize NLP techniques to handle different informant-based information. These steps will enhance the robustness and accuracy of semantic embeddings in data harmonization. In this respect, it is also important to note the importance of developing clear and comprehensive data dictionaries [58] and/or making use of existing ones and integrating the respective information in the harmonization process. This is not only essential for a concrete overview of data sets from different cohorts but also for planning future studies in alignment with those cohorts that might be most important for potential later cross-cohort analyses. Our preliminary results encourage future research, including model fine-tuning and applications on larger texts or metadata elements, aiming to develop tools for semantic and syntactic mapping in metadata repositories and automate the harmonization process.

## Limitations

While our investigation establishes a fundamental principle for utilizing embeddings in cohort study harmonizations for similarity searches and underscores and expands recent studies in this context (e.g., [17]), this research is still in its early stages. The efficiency and potential of this approach in evaluating harmonization opportunities are significant strengths, which have been tested in a larger number of questionnaires and potential pairs (here: over 70,000) than in previous applications [17] as well as now based on item texts from different languages. However, the user perspective, including input and additional evaluations from researchers and experts, should not be excluded from this process. For this reason, we have incorporated multiple layers into the “Semantic Search Helper,” drawing on information from standard terminologies in the field and allowing data adjustments at various levels to enable customized visualization. However, further examination and testing are necessary to fully appreciate its potential. Intercultural and historical effects on item formulation can influence item semantics, meaning identical formulations can differ across cultures or eras. These differences are not fully captured by semantic embeddings, posing a limitation for automated data harmonization algorithms. Despite its limitations, our research validates this approach’s efficacy and establishes a foundation for further exploration.

Our goal was to strengthen cross-cohort and -study based research in mental health by simplifying the time-intensive process of data harmonization through scalable, NLP-based methods. NLP provides an efficient way to identify harmonization potential and conserve resources, that should however not be treated independently from available standard sources and manual processes and the researchers’ expertise. This methodology can thus not completely replace human expertise and the nuanced semantic assessments, as our work demonstrates. In the long term, this research aims to establish pooling – combining data from different studies – as a common method to increase sample sizes and thereby strengthen the validity of scientific analyses.

Together, this research demonstrates that attention-based embeddings are effective for identifying semantic similarities and can thus assist researchers in this task. Using these techniques in questionnaire-based cohort data provides a viable approach for the initial phases of data harmonization and merits further investigation. Our code is available on GitHub under the MIT license upon request. The decision to release it upon request is driven by our goal to use user feedback, gathered through an evaluation questionnaire, to improve and adapt the prototype. Users can also submit suggestions for future iterations.

## Supporting information

Gottfried et al. supplementary material Gottfried et al. supplementary material

## Data Availability

The data underlying this article cannot be shared publicly due to ethical reasons. The data will be shared on reasonable request to the corresponding author.

## Abbreviations

ADA: Name of a specific model or method used in the context
ADHS-LE: ADHS-Langzeit-Erfassung
ADS: Alcohol Dependence Scale
AID: Adaptives Intelligenz Diagnostikum
APQ-2: Advanced Personality Questionnaire second version
AUDIT: Alcohol Use Disorders Identification Test
BDI: Beck Depression Inventory
BelVa: Belgian Violent behavior Assessment
BERT: Bidirectional Encoder Representations from Transformers
CBQ: Comprehensive Behavioral Questionnaire
EBSK: Eltern-Belastungs-Screening
EPDS: Edinburgh Postnatal Depression Scale
ESI: Environmental Stress Index
EtG: Ethyl glucuronide
FBB-ADHS: ADHD-specific Behavior Assessment Form
FBB-ANZ: Anxiety-specific Behavior Assessment Form
FBB-DES: Depression-specific behavior assessment form
FBB-SSV: Social Behavior Assessment Form
FRANCES: Franconian Cognition and Emotion Studies
FTND: Fagerström Test for Nicotine Dependence
IDS: Inventory of Depressive Symptomatology
IMAC-Mind: Improving mental health and reducing addiction in childhood and adolescence through mindfulness
KiGa: Kindergarten Adjustment Scale
KINDL: KINDL questionnaire for measuring health-related quality of life in children and adolescents
LES: Life Experiences Survey
LOINC: Logical Observation Identifiers Names and Codes
MARS: Mannheim Study of Children at Risk
MDM: Medical data models
NLP: Natural language processing
NEO-FFI: NEO Personality Inventory
POSEIDON: Pre- peri- and postnatal stress: epigenetic impact on depression
PSS: Perceived Stress Scale
ROLS: Rostocker Längsschnittstudie
SBERT: Sentence-BERT
SDQ: Strengths and Difficulties Questionnaire
STAI-S: State-Trait Anxiety Inventory – short form
STAI-T: State-Trait Anxiety Inventory – trait form
SVF: Stress Coping Inventory
TF-IDF: Frequency-inverse document frequency
TPF: Typical Personality Factors Questionnaire
UMAP: Uniform manifold approximation and projection

## Supplementary material

The supplementary material for this article can be found at https://doi.org/10.1192/j.eurpsy.2024.1808.
