# Supplementary material for: Semantic search helper: A tool based on the use of embeddings in multi-item questionnaires as a harmonization opportunity for merging large datasets – A feasibility study
Source: Eur Psychiatry. 2025 Jan 20;68(1):e8. doi: 10.1192/j.eurpsy.2024.1808 (PMC11795448; doi:10.1192/j.eurpsy.2024.1808)
Supplement: Gottfried et al. supplementary material [file S092493382401808Xsup001.docx]

**Supplementary data**

**Table S1**: List of topics generated by Semantic Search Helper (using BERTopic [65]) during the embedding building step.

| **Topic** | **Representation** | **Count** |
| --- | --- | --- |
| 0 | to,and,the,my,in,or,of,it,often,me | 1421 |
| 1 | you,drinking,alcohol,have,how,year,last,during,often,been | 21 |
| 2 | smoke,you,do,cigarettes,smoking,first,tobacco,day,how,cigarette | 16 |
|  |  | **1458** |

**Table S2**: Filtered item pairs with a similarity score greater than 0.75 generated by Semantic Search Helper during the exploration step (sorted by similarity score).

| **Questionnaire 1** | **Item 1** | **Questionnaire 2** | **Item 2** | **Similarity score** |
| --- | --- | --- | --- | --- |
| NEO-FFI (long version) | I often feel tense and nervous. | NEO-FFI (short version) | I often feel tense and nervous. | 0.9999999404 |
| NEO-FFI (long version) | I often feel like I'm overflowing with energy | NEO-FFI (short version) | I often feel like I'm overflowing with energy. | 0.9967021346 |
| NEO-FFI (long version) | I often get into arguments with my family or colleagues. | NEO-FFI (short version) | I frequently get into arguments with my family and colleagues. | 0.9937576652 |
| NEO-FFI (long version) | Sometimes, when I read literature or view artwork, I experience a shiver or a wave of enthusiasm. | NEO-FFI (short version) | When I read literature or observe an artwork, I sometimes feel a shiver or a wave of enthusiasm. | 0.9927321076 |
| NEO-FFI (long version) | All too often, I'm discouraged and want to give up when something goes wrong. | NEO-FFI (short version) | Too often, I get discouraged and want to give up when something goes wrong. | 0.984085083 |
| NEO-FFI (long version) | When I am under severe stress, I sometimes feel like I am falling apart. | NEO-FFI (short version) | When I'm under intense stress, I sometimes feel like I'm falling apart. | 0.9835675955 |
| ADHS-LE | It's not easy to disturb my peace. | NEO-FFI (long version) | It's not easy to disturb my peace | 0.980973959 |
| PSS | I enjoy playing pranks on others. | Social support questionnaire | I really enjoy playing pranks on others. | 0.9780694246 |
| NEO-FFI (long version) | I will probably never be able to bring order to my life. | NEO-FFI (short version) | I will probably never be capable of bringing order to my life. | 0.9769996405 |
| EBSK | I often feel worthless. | NEO-FFI (long version) | Sometimes I feel completely worthless. | 0.9673119187 |
| EBSK | I often feel worthless. | NEO-FFI (short version) | Sometimes I feel completely worthless. | 0.9673119187 |
| NEO-FFI (long version) | I often feel inferior to others. | TPF | I feel inferior to other people. | 0.9657915831 |
| TPF | I feel inferior to other people. | NEO-FFI (short version) | I often feel inferior to others. | 0.9657915831 |
| NEO-FFI (long version) | Some people consider me selfish and self-centered. | NEO-FFI (short version) | Some people consider me selfish and self-indulgent. | 0.9632600546 |
| EBSK | I often feel lonely. | TPF | I feel lonely. | 0.962434411 |
| NEO-FFI (long version) | I am a cheerful, good-natured person. | NEO-FFI (short version) | I am a cheerful, good-humored person. | 0.9612823725 |
| NEO-FFI (long version) | When it comes to the intentions of others, I am rather cynical and skeptical. | NEO-FFI (short version) | I tend to be cynical and skeptical about others' intentions. | 0.9557270408 |
| NEO-FFI (long version) | I try to fulfill all tasks entrusted to me very conscientiously. | NEO-FFI (short version) | I try to complete all tasks assigned to me very conscientiously. | 0.9472811222 |
| STAI-S | I am happy. | STAI-T | I am cheerful. | 0.9450948238 |
| STAI-S | I am cheerful. | STAI-T | I am happy. | 0.9450947642 |
| NEO-FFI (long version) | I am a capable person who always gets their work done. | NEO-FFI (short version) | I am a capable person who always completes their work. | 0.9389098883 |
| NEO-FFI (long version) | I often feel helpless and wish for a person who can solve my problems. | NEO-FFI (short version) | I feel helpless and wish for someone to solve my problems. | 0.9378631711 |
| EBSK | I often feel lonely. | Social support questionnaire | I often feel lonely even when people are around me. | 0.9282821417 |
| CBQ | Is afraid of the dark. | PSS | I'm afraid of the dark. | 0.9232852459 |
| AID | Achievement Motivation | IDS | Achievement Motivation Score | 0.9231746197 |
| NEO-FFI (long version) | I keep my things neat and clean. | NEO-FFI (short version) | I keep my things organized and clean. | 0.9211779833 |
| NEO-FFI (long version) | I avoid crowds of people. | TPF | I avoid big crowds and commotions. | 0.9178020954 |
| STAI-S | I am calm. | STAI-T | I am calm and composed. | 0.9165526628 |
| AID | Fine Motor Skills | IDS | Fine Motor Skills Score | 0.9124588966 |
| ESI | Struggles to get along with family. | EBSK | My family has trouble getting along. | 0.908597827 |
| NEO-FFI (long version) | I tend to blame myself when something goes wrong. | Social support questionnaire | When I've done something wrong, I often try to blame others for it. | 0.9072347879 |
| PSS | I have trouble sleeping at night and wake up frequently. | ADS | I have trouble sleeping at night | 0.9065966606 |
| NEO-FFI (long version) | I often had a leadership position in groups I belonged to. | TPF | In a group, I take on a leadership role. | 0.9035010338 |
| EBSK | I am often internally lonely. | Social support questionnaire | I often feel lonely even when people are around me. | 0.9028046727 |
| NEO-FFI (long version) | I usually prefer to do things alone. | Social support questionnaire | I prefer to be alone. | 0.9002023935 |
| NEO-FFI (long version) | I am easily scared. | PSS | I am easily frightened by something. | 0.8996053934 |
| AID | Gross Motor Skills | IDS | Gross Motor Skills Score | 0.8985663652 |
| EBSK | I am often internally lonely. | TPF | I feel lonely. | 0.8968113661 |
| SVF | I'm depressed | EBSK | I am often depressed. | 0.8958412409 |
| ADHS-LE | Before I act, I think about the consequences. | NEO-FFI (long version) | Before I act, I generally consider the possible consequences | 0.8953450918 |
| Smoking habit interview | How many cigarettes does your husband smoke on average daily? | FTND | How many cigarettes do you smoke per day? | 0.8841600418 |
| NEO-FFI (short version) | I am a cheerful, good-humored person. | STAI-S | I am cheerful. | 0.8824718595 |
| NEO-FFI (short version) | I am a cheerful, good-humored person. | STAI-T | I am cheerful. | 0.8824718595 |
| TPF | I feel lonely. | Social support questionnaire | I often feel lonely even when people are around me. | 0.8773337603 |
| NEO-FFI (long version) | I am easy to make laugh. | NEO-FFI (short version) | I'm easily amused and can laugh easily. | 0.8772482872 |
| NEO-FFI (long version) | I often find it hard to make decisions. | PSS | Often I find it difficult to decide what to do. | 0.8729008436 |
| FBB-DES | Appears restless, tense, and driven most of the time. | FBB-ANZ | Appears nervous, restless, overactive, constantly tense, and strained. | 0.8706240058 |
| NEO-FFI (long version) | Most people I know like me. | TPF | I feel that most people like me. | 0.8670873642 |
| EBSK | I often feel anxious. | NEO-FFI (long version) | I often feel tense and nervous. | 0.8668384552 |
| EBSK | I often feel anxious. | NEO-FFI (short version) | I often feel tense and nervous. | 0.8668384552 |
| TPF | I behave in a way that people could see me as selfish. | NEO-FFI (short version) | Some people consider me selfish and self-indulgent. | 0.8642576337 |
| FBB-SSV | Frequently blames others for their own mistakes or misbehavior. | Social support questionnaire | When I've done something wrong, I often try to blame others for it. | 0.8554186821 |
| STAI-S | I am happy. | STAI-T | I am content. | 0.8541124463 |
| STAI-S | I am content. | STAI-T | I am happy. | 0.8541123867 |
| NEO-FFI (short version) | When I'm under intense stress, I sometimes feel like I'm falling apart. | ADS | I feel like I'm falling apart and going to pieces | 0.854069829 |
| NEO-FFI (long version) | Some people consider me selfish and self-centered. | TPF | I behave in a way that people could see me as selfish. | 0.8520182967 |
| SVF | I'd rather be alone | Social support questionnaire | I prefer to be alone. | 0.8508227468 |
| NEO-FFI (long version) | When I am under severe stress, I sometimes feel like I am falling apart. | ADS | I feel like I'm falling apart and going to pieces | 0.8507785797 |
| PSS | I believe I am more nervous than others. | ADS | I feel more nervous and anxious than usual | 0.8477184772 |
| STAI-S | I am content. | STAI-T | I am cheerful. | 0.8466824889 |
| STAI-S | I am cheerful. | STAI-T | I am content. | 0.8466824889 |
| TPF | I am a calm and balanced person. | STAI-T | I am calm and composed. | 0.845541358 |
| NEO-FFI (long version) | I can manage my time fairly well, so that I finish my affairs on time. | NEO-FFI (short version) | I can manage my time quite well to complete my tasks on time. | 0.845197916 |
| ADHS-LE | It's hard for me to motivate myself for a task. | NEO-FFI (long version) | I struggle to motivate myself to do what I should | 0.8446161151 |
| AID | Achievement Motivation | IDS | Achievement Motivation Average Score | 0.8438265324 |
| NEO-FFI (long version) | I often find it hard to make decisions. | PSS | It's hard for me to make decisions about anything. | 0.842539072 |
| FBB-DES | Has little appetite most of the time. | PSS | I often have a poor appetite. | 0.8424630165 |
| NEO-FFI (long version) | When I do something, I do it with a lot of enthusiasm. | TPF | I am full of enthusiasm, almost exuberant. | 0.8397521377 |
| ADHS-LE | When someone annoys me, I often say things I later regret. | PSS | I often do things that I later regret. | 0.8363680243 |
| NEO-FFI (long version) | At times, I have been angry and embittered. | PSS | Others often provoke me until I get angry. | 0.8356906176 |
| ADHS-LE | I blurt out answers before the question has been fully asked. | FBB-ADHS | Often blurts out answers before questions have been fully asked. | 0.8355610967 |
| PSS | I believe I am more nervous than others. | STAI-S | I am nervous. | 0.8346613646 |
| EBSK | I often feel very alone. | Social support questionnaire | I often feel lonely even when people are around me. | 0.8344109654 |
| ADHS-LE | In stressful moments, I usually keep a cool head. | NEO-FFI (long version) | In emergencies, I keep a cool head | 0.8341554403 |
| EBSK | My life is happy. | STAI-T | I am happy. | 0.8340419531 |
| EBSK | My life is happy. | STAI-S | I am happy. | 0.8340419531 |
| SVF | I can't see why I always have to be unlucky | EBSK | I am always unlucky. | 0.8340107799 |
| EPDS | I was so unhappy that I had to cry. | STAI-T | I feel like crying. | 0.8338381052 |
| SVF | I avoid people | NEO-FFI (long version) | I avoid crowds of people. | 0.8330064416 |
| ADHS-LE | I get angry too quickly. | FBB-SSV | Becomes angry quickly. | 0.8319571018 |
| NEO-FFI (long version) | I am often worried about things that could go wrong. | STAI-T | I worry about possible mishaps. | 0.8301833868 |
| Smoking habit interview | How many cigarettes did you smoke daily until admission to the clinic? | FTND | How many cigarettes do you smoke per day? | 0.8283164501 |
| NEO-FFI (long version) | I am often worried about things that could go wrong. | PSS | I often fear that something bad could happen. | 0.8281090856 |
| STAI-S | I am sad. | STAI-T | I feel like crying. | 0.8277928829 |
| STAI-S | I am worried that something might go wrong. | STAI-T | I worry about possible mishaps. | 0.827341795 |
| NEO-FFI (long version) | I try to always be considerate and sensitive | NEO-FFI (short version) | I always try to act considerately and sensitively. | 0.8271405697 |
| NEO-FFI (long version) | I am often worried about things that could go wrong. | STAI-S | I am worried that something might go wrong. | 0.825158 |
| ADHS-LE | I get angry too quickly. | SVF | I get angry | 0.8246392012 |
| ADHS-LE | I consider myself a balanced person. | STAI-T | I am well-balanced. | 0.8246044517 |
| EBSK | I often feel very alone. | TPF | I feel lonely. | 0.8241876364 |
| NEO-FFI (long version) | I am a cheerful, good-natured person. | STAI-S | I am cheerful. | 0.8238110542 |
| NEO-FFI (long version) | I am a cheerful, good-natured person. | STAI-T | I am cheerful. | 0.8238110542 |
| NEO-FFI (long version) | I often feel tense and nervous. | STAI-S | I feel tense. | 0.8209247589 |
| NEO-FFI (short version) | I often feel tense and nervous. | STAI-S | I feel tense. | 0.8209247589 |
| SVF | I tend to clash with other people | PSS | I often simply feel like fighting with others. | 0.8197390437 |
| PSS | I like to make fun of other kids who are not as good as me or laugh at them. | Social support questionnaire | I feel that other children often laugh at me. | 0.819674015 |
| Alcohol consumption Interview | Wie oft waren Sie im letzten halben Jahr betrunken? | AUDIT | How often do you have a drink containing alcohol? | 0.8192367554 |
| ADHS-LE | I get distracted by things happening around me. | SVF | I somehow distract myself | 0.8185515404 |
| TPF | Mental health | LES | Major Mental Crisis | 0.8176465034 |
| CBQ | Is afraid of loud noises. | FBB-ANZ | Has a pronounced and persistent fear of loud noises that is exaggerated and unjustified (e.g. popping sounds or thunder). | 0.8157241344 |
| TPF | I am full of enthusiasm, almost exuberant. | STAI-S | I am excited. | 0.8156763315 |
| PSS | Sometimes I boast a little. | Social support questionnaire | When I'm with others, I often boast. | 0.8152140379 |
| SVF | I get angry | PSS | Others often provoke me until I get angry. | 0.814920485 |
| FBB-SSV | Frequently blames others for their own mistakes or misbehavior. | NEO-FFI (long version) | I tend to blame myself when something goes wrong. | 0.8142434359 |
| SVF | I become irritated | EBSK | I often get agitated. | 0.8141462803 |
| SVF | I blame myself | NEO-FFI (long version) | I tend to blame myself when something goes wrong. | 0.8139048219 |
| SVF | I blame myself | NEO-FFI (long version) | I tend to blame myself when something goes wrong. | 0.8139048219 |
| NEO-FFI (long version) | I often feel tense and nervous. | ADS | I feel more nervous and anxious than usual | 0.8132839203 |
| NEO-FFI (short version) | I often feel tense and nervous. | ADS | I feel more nervous and anxious than usual | 0.8132839203 |
| TPF | I feel like everything is too much for me. | EPDS | Everything became too much for me. | 0.8122107983 |
| STAI-S | I am excited. | STAI-T | I am happy. | 0.8121615052 |
| NEO-FFI (long version) | I often feel tense and nervous. | ADS | I get upset easily or feel panicky | 0.8121238351 |
| NEO-FFI (short version) | I often feel tense and nervous. | ADS | I get upset easily or feel panicky | 0.8121238351 |
| PSS | Sometimes I shout at other people when I'm upset. | Social support questionnaire | I sometimes yell at adults when I'm angry. | 0.8109323978 |
| CBQ | Has difficulty concentrating during an activity. | FBB-ADHS | Often has difficulty sustaining attention in tasks or play activities. | 0.8107321262 |
| EBSK | Many things in life make me angry. | PSS | Others often provoke me until I get angry. | 0.8106569648 |
| NEO-FFI (long version) | I often find it hard to make decisions. | TPF | It is difficult for me to make decisions independently. | 0.8101218939 |
| TPF | I am a calm and balanced person. | STAI-S | I am calm. | 0.8097083569 |
| PSS | I become restless and nervous quickly if things don't go as I'd like. | STAI-T | I become nervous and restless when I think about my current affairs. | 0.8075328469 |
| ADHS-LE | I struggle to organize the completion of multiple tasks. | FBB-ADHS | Often encounters difficulty organizing tasks and activities. | 0.8070875406 |
| PSS | I get easily upset when someone scolds or criticizes me. | Social support questionnaire | I often have the desire to annoy others or argue with them. | 0.806733489 |
| FBB-DES | Has lost a significant amount of weight recently without dieting. | ADS | I have lost weight recently without trying to do so | 0.8064088225 |
| EBSK | I often get agitated. | PSS | Others often provoke me until I get angry. | 0.8053560257 |
| SVF | I feel guilty | BDI | Guilty feelings | 0.8048251867 |
| TPF | I feel that most people like me. | Social support questionnaire | Other people can like me as well as my friends do. | 0.8033542633 |
| EBSK | Many things in life make me angry. | NEO-FFI (long version) | At times, I have been angry and embittered. | 0.8030983806 |
| EBSK | I often feel anxious. | ADS | I feel more nervous and anxious than usual | 0.8025813103 |
| FBB-SSV | Often starts fights with other children. | SDQ | Often argues with or bullies other children | 0.8024990559 |
| NEO-FFI (short version) | I am a cheerful, good-humored person. | STAI-S | I am happy. | 0.8023972511 |
| NEO-FFI (short version) | I am a cheerful, good-humored person. | STAI-T | I am happy. | 0.8023971915 |
| FBB-DES | Has trouble sleeping most nights or almost every night. | PSS | I have trouble sleeping at night and wake up frequently. | 0.8021569848 |
| STAI-S | I am uneasy. | ADS | I have a feeling of dread | 0.8018874526 |
| AID | Gross Motor Skills | IDS | Fine Motor Skills Score | 0.8016486168 |
| EBSK | I often feel anxious. | ADS | I have a feeling of dread | 0.7990325689 |
| CBQ | Cries even with minor injuries. | BDI | Crying | 0.7985287905 |
| Alcohol consumption Interview | Wie oft waren Sie im letzten halben Jahr betrunken? | AUDIT | How often during the last year have you found that you were not able to stop drinking once you had started? | 0.7983354926 |
| EBSK | I often get agitated. | PSS | I tend to get upset quickly about something. | 0.7976954579 |
| NEO-FFI (long version) | I usually prefer to do things alone. | Social support questionnaire | I prefer to spend my free time alone rather than with others. | 0.7976498604 |
| ADHS-LE | My affection is often overwhelming for others. | TPF | Showing feelings of affection and tenderness is difficult for me. | 0.7975151539 |
| NEO-FFI (long version) | Poetry doesn't impress me much or at all. | NEO-FFI (short version) | Poetry impresses me little or not at all. | 0.797445178 |
| ESI | Disagreements with others about parenting. | APQ-2 | You find it difficult to be consistent in your parenting. | 0.7958475947 |
| PSS | I like to make fun of other kids who are not as good as me or laugh at them. | Social support questionnaire | I'm often teased or mocked by other children. | 0.7953976393 |
| EBSK | I am usually a quiet person. | Social support questionnaire | I'm usually quiet and don't say anything when I'm with others. | 0.7951934934 |
| ADHS-LE | I'm often under stress or inner tension. | NEO-FFI (short version) | When I'm under intense stress, I sometimes feel like I'm falling apart. | 0.7950542569 |
| TPF | I am convinced that people can like me a lot. | Social support questionnaire | Other people can like me as well as my friends do. | 0.7944110632 |
| EBSK | I often get agitated. | NEO-FFI (long version) | At times, I have been angry and embittered. | 0.794064045 |
| EBSK | Sometimes I feel very alone in this world. | Social support questionnaire | I often feel lonely even when people are around me. | 0.7937905192 |
| PSS | I often feel bored and don't know what to do with myself. | TPF | I get bored. | 0.7934485078 |
| ADHS-LE | After a busy day, I have trouble relaxing. | EBSK | I find it hard to relax. | 0.7928839326 |
| NEO-FFI (long version) | It takes a lot to get me upset. | PSS | I tend to get upset quickly about something. | 0.7908697128 |
| ADHS-LE | I get angry too quickly. | PSS | I tend to get upset quickly about something. | 0.7903578877 |
| PSS | I enjoy mocking or ridiculing others. | Social support questionnaire | I like it when other children or adults can laugh at my jokes and antics. | 0.7894945145 |
| ADHS-LE | I get angry too quickly. | FBB-SSV | Frequently angry and gets upset quickly. | 0.7890185714 |
| AID | Fine Motor Skills | IDS | Gross Motor Skills Score | 0.7889056206 |
| SVF | I'm dissatisfied with myself | EBSK | I am often internally frustrated. | 0.7887555957 |
| SVF | I get angry | NEO-FFI (long version) | At times, I have been angry and embittered. | 0.7879219651 |
| SVF | I tend to give up | NEO-FFI (short version) | Too often, I get discouraged and want to give up when something goes wrong. | 0.7878376245 |
| EBSK | My family argues a lot. | NEO-FFI (short version) | I frequently get into arguments with my family and colleagues. | 0.7865269184 |
| ADHS-LE | Sometimes I feel helpless against my feelings. | PSS | Sometimes I feel "miserable" without any specific reason. | 0.7864719629 |
| SVF | I blame myself | Social support questionnaire | When I've done something wrong, I often try to blame others for it. | 0.7862317562 |
| SVF | I blame myself | Social support questionnaire | When I've done something wrong, I often try to blame others for it. | 0.7862317562 |
| ADHS-LE | I get distracted by things happening around me. | PSS | I easily get distracted in class by other things. | 0.7856847048 |
| NEO-FFI (long version) | Sometimes I pester people or flatter them to get them to do what I want. | Social support questionnaire | I feel that others sometimes do with me what they want. | 0.7854310274 |
| EBSK | I often feel anxious. | STAI-T | I become nervous and restless when I think about my current affairs. | 0.7854064107 |
| FBB-SSV | Frequently angry and gets upset quickly. | SVF | I get angry | 0.7850129604 |
| EBSK | I often get agitated. | ADS | I get upset easily or feel panicky | 0.7849394679 |
| PSS | I am often so restless that I can't sit still for long on a chair. | ADS | I am restless and can't keep still | 0.7848852873 |
| PSS | Others often provoke me until I get angry. | Social support questionnaire | I sometimes yell at adults when I'm angry. | 0.7848154902 |
| STAI-S | I am relaxed. | STAI-T | I feel rested. | 0.7845653296 |
| NEO-FFI (long version) | Sometimes I influence people in such a way that they do what I want. | Social support questionnaire | I feel that others sometimes do with me what they want. | 0.7845288515 |
| FBB-DES | Cries a lot. | BDI | Crying | 0.784507513 |
| SVF | I get angry | EBSK | Many things in life make me angry. | 0.7843824029 |
| PSS | Others often provoke me until I get angry. | Social support questionnaire | I often have the desire to annoy others or argue with them. | 0.7843081951 |
| FBB-DES | Appears grumpy, irritable, and in a bad mood most of the time. | FBB-ANZ | Appears nervous, restless, overactive, constantly tense, and strained. | 0.7842856646 |
| TPF | I feel that most people like me. | Social support questionnaire | I feel that people like me as much as other children. | 0.7840676308 |
| EBSK | I am often internally frustrated. | NEO-FFI (long version) | At times, I have been angry and embittered. | 0.7836934328 |
| SDQ | Constantly fidgety | STAI-S | I am fidgety. | 0.7822019458 |
| NEO-FFI (long version) | I often get annoyed about how other people treat me. | Social support questionnaire | I often have the desire to annoy others or argue with them. | 0.7816499472 |
| EBSK | Life often seems useless to me. | TPF | The thought that my life could be meaningless has occurred to me. | 0.7816417813 |
| FBB-SSV | Frequently angry and gets upset quickly. | PSS | Others often provoke me until I get angry. | 0.7809251547 |
| EPDS | I was sad and felt miserable. | STAI-T | I feel like crying. | 0.7804043293 |
| ADHS-LE | I consider myself a balanced person. | NEO-FFI (long version) | I am a person with a balanced temperament. | 0.779902935 |
| SVF | I avoid such situations from now on | TPF | I tend to avoid uncomfortable situations. | 0.7798271179 |
| EBSK | My life is happy. | STAI-S | I am cheerful. | 0.7797373533 |
| EBSK | My life is happy. | STAI-T | I am cheerful. | 0.7797373533 |
| EBSK | I often feel very unsettled. | ADS | I have a feeling of dread | 0.779686451 |
| EBSK | I often get upset and don't know why. | PSS | I tend to get upset quickly about something. | 0.7796186209 |
| ESI | Caring for other family members. | LES | Caring for / Nursing a Family Member (>3 Months) | 0.7796084881 |
| KiGa | Is the mood cheerful? | TPF | My mood is good. | 0.7793303728 |
| EBSK | Sometimes I feel very alone in this world. | TPF | I feel lonely. | 0.7792967558 |
| FBB-SSV | Becomes angry quickly. | SVF | I get angry | 0.7791355848 |
| TPF | I worry a lot. | STAI-S | I am concerned. | 0.7780769467 |
| FBB-DES | Has an excessive appetite most of the time. | BDI | Changes in appetite | 0.7779493332 |
| EBSK | I often get agitated. | NEO-FFI (long version) | I often feel tense and nervous. | 0.7771906257 |
| EBSK | I often get agitated. | NEO-FFI (short version) | I often feel tense and nervous. | 0.7771906257 |
| ADHS-LE | I get distracted by things happening around me. | SVF | I do something to distract myself | 0.777035594 |
| EBSK | I often feel lonely. | NEO-FFI (long version) | I rarely feel lonely or sad | 0.7764353752 |
| ADHS-LE | My mood frequently changes within a few minutes. | CBQ | Changes from sad to happier mood within minutes. | 0.7746602297 |
| LES | Suicide Attempt | BDI | Suicidal thoughts or wishes | 0.7738910913 |
| PSS | Sometimes I shout at other people when I'm upset. | Social support questionnaire | I often have the desire to annoy others or argue with them. | 0.7736981511 |
| TPF | In a group, I take on a leadership role. | Social support questionnaire | Among my friends, I'm often the leader when we do things together. | 0.773491919 |
| SDQ | Restless, hyperactive | FBB-DES | Appears restless, tense, and driven most of the time. | 0.7732121348 |
| SVF | I think about how I can avoid such situations from now on | TPF | I tend to avoid uncomfortable situations. | 0.7730983496 |
| SVF | I tend to give up | NEO-FFI (long version) | All too often, I'm discouraged and want to give up when something goes wrong. | 0.7728589773 |
| ADHS-LE | I struggle to organize the completion of multiple tasks. | FBB-ADHS | Often struggles to complete tasks or assignments and frequently leaves them unfinished. | 0.7728180885 |
| ESI | Struggles to get along with family. | LES | Severe Conflicts with Family (>3 Months) | 0.7727937698 |
| PSS | I get excited about things quickly. | TPF | I am full of enthusiasm, almost exuberant. | 0.7724975944 |
| NEO-FFI (long version) | I am known as a warm and friendly person. | NEO-FFI (short version) | I am a cheerful, good-humored person. | 0.7714037895 |
| ADHS-LE | I'm often under stress or inner tension. | EBSK | I am often internally frustrated. | 0.7713674307 |
| EBSK | I often feel anxious. | ADS | I get upset easily or feel panicky | 0.771356225 |
| EBSK | My family argues a lot. | NEO-FFI (long version) | I often get into arguments with my family or colleagues. | 0.7711447477 |
| PSS | I feel that many people don't have good intentions towards me. | Social support questionnaire | I feel that others don't agree with how I approach things. | 0.7699176669 |
| STAI-T | I feel like crying. | BDI | Crying | 0.7697095871 |
| SDQ | Often complains of headaches, stomachaches, and nausea | PSS | I have headaches very often. | 0.7695689797 |
| ADHS-LE | I consider myself a balanced person. | TPF | I am a calm and balanced person. | 0.7694860697 |
| EBSK | I often feel anxious. | STAI-S | I am uneasy. | 0.7690207958 |
| FBB-SSV | Is easily annoyed or provoked by others. | PSS | I get easily upset when someone scolds or criticizes me. | 0.7681549788 |
| NEO-FFI (long version) | I often feel tense and nervous. | STAI-T | I become nervous and restless when I think about my current affairs. | 0.767996192 |
| NEO-FFI (short version) | I often feel tense and nervous. | STAI-T | I become nervous and restless when I think about my current affairs. | 0.767996192 |
| PSS | I enjoy playing pranks on others. | Social support questionnaire | I like it when other children or adults can laugh at my jokes and antics. | 0.7674070001 |
| ADHS-LE | When I lie in bed at night, I find it hard to relax. | EBSK | I find it hard to relax. | 0.7672230005 |
| NEO-FFI (long version) | I often get annoyed about how other people treat me. | Social support questionnaire | I get annoyed when people don't do what I want. | 0.766078651 |
| STAI-S | I feel confident. | STAI-T | I feel secure. | 0.7657699585 |
| NEO-FFI (long version) | I often get annoyed about how other people treat me. | PSS | Sometimes I speak badly about other people. | 0.7656239867 |
| CBQ | Often laughs loudly when playing with other children. | Social support questionnaire | I feel that other children often laugh at me. | 0.7649883032 |
| ADHS-LE | Sometimes I feel helpless against my feelings. | TPF | A feeling of powerlessness and helplessness overwhelms me. | 0.7647846937 |
| TPF | My heart starts racing or stumbling and beating irregularly. | ADS | My heart beats faster than usual | 0.7646717429 |
| NEO-FFI (long version) | When I do something, I do it with a lot of enthusiasm. | PSS | I get excited about things quickly. | 0.7642934322 |
| EBSK | I often feel very frustrated. | NEO-FFI (long version) | At times, I have been angry and embittered. | 0.7634290457 |
| EPDS | I was sad and felt miserable. | STAI-S | I am sad. | 0.7631543875 |
| CBQ | Is afraid of things like animals or the dark. | PSS | I'm afraid of the dark. | 0.762984395 |
| TPF | Self-esteem | BDI | Self-criticalness | 0.7625747919 |
| ADHS-LE | I'm often under stress or inner tension. | NEO-FFI (long version) | When I am under severe stress, I sometimes feel like I am falling apart. | 0.762327373 |
| TPF | Self-esteem | BDI | Self-dislike | 0.762174964 |
| Alcohol consumption Interview | Wie oft waren Sie im letzten halben Jahr betrunken? | AUDIT | How often during the last year have you failed to do what was normally expected from you because of drinking? | 0.7620877624 |
| NEO-FFI (long version) | I often feel inferior to others. | TPF | I feel misunderstood by other people. | 0.7620013952 |
| TPF | I feel misunderstood by other people. | NEO-FFI (short version) | I often feel inferior to others. | 0.7620013952 |
| SVF | I become irritated | BDI | Irritability | 0.7618124485 |
| NEO-FFI (long version) | I find it difficult to take on a leadership role. | TPF | In a group, I take on a leadership role. | 0.7617338896 |
| FBB-DES | Has trouble sleeping most nights or almost every night. | ADS | I have trouble sleeping at night | 0.7616331577 |
| FBB-SSV | Frequently angry and gets upset quickly. | EBSK | I often get agitated. | 0.7613732219 |
| TPF | I feel inferior to other people. | STAI-T | I believe I am worse off than other people. | 0.7611234188 |
| SVF | Everything seems so hopeless to me | NEO-FFI (long version) | Sometimes everything seems quite dark and hopeless to me. | 0.7608673573 |
| AID | Independent | TPF | Autonomy | 0.7607927918 |
| NEO-FFI (long version) | Sometimes frightening thoughts come to my mind. | STAI-T | Unimportant thoughts go through my mind and trouble me. | 0.7607761025 |
| AID | Basic Mood | KiGa | Is the mood cheerful? | 0.7603391409 |
| STAI-T | I become tired quickly. | ADS | I get tired for no reason | 0.7602670789 |
| NEO-FFI (long version) | Most people I know like me. | Social support questionnaire | Other people can like me as well as my friends do. | 0.7597204447 |
| NEO-FFI (long version) | I am easily scared. | PSS | Secretly, I'm afraid of many things. | 0.7596399188 |
| EBSK | I often get upset and don't know why. | NEO-FFI (long version) | It takes a lot to get me upset. | 0.7596324086 |
| ADHS-LE | I complete tasks I start. | NEO-FFI (short version) | I try to complete all tasks assigned to me very conscientiously. | 0.7590005398 |
| TPF | I feel ill. | STAI-S | I feel tense. | 0.7588819265 |
| SVF | I tend to clash with other people | Social support questionnaire | I often have the desire to annoy others or argue with them. | 0.75882411 |
| TPF | I am a calm and balanced person. | STAI-T | I am well-balanced. | 0.7585830092 |
| Alcohol consumption Interview | Wie oft waren Sie im letzten halben Jahr betrunken? | AUDIT | How often during the last year have you needed a first drink in the morning to get yourself going after a heavy drinking session? | 0.7584298849 |
| STAI-S | I feel comfortable. | STAI-T | I feel secure. | 0.7580180168 |
| EBSK | Things in my life have usually gone against me. | TPF | Things go wrong for me. | 0.7577058077 |
| ADHS-LE | I have deliberately caused myself pain or intentionally harmed myself. | EBSK | A lot of pain has been inflicted on me. | 0.7575637102 |
| ADHS-LE | People who know me well describe me as suspicious. | NEO-FFI (long version) | I become suspicious when someone does me a favor. | 0.7575138807 |
| NEO-FFI (long version) | I often get annoyed about how other people treat me. | Social support questionnaire | Others are often mean to me. | 0.7574243546 |
| LES | Suicide Attempt / Crisis of Family Member/Friend | BDI | Suicidal thoughts or wishes | 0.7572641373 |
| NEO-FFI (long version) | Most people I know like me. | TPF | I am convinced that people can like me a lot. | 0.7569354773 |
| AID | Articulation | BDI | Agitation | 0.7568380237 |
| PSS | It's hard for me to make decisions about anything. | TPF | It is difficult for me to make decisions independently. | 0.7561687231 |
| NEO-FFI (long version) | I enjoy lively parties. | Social support questionnaire | In a fun gathering, like a birthday party, I can relax and be cheerful. | 0.7557496428 |
| STAI-S | I feel tense. | STAI-T | I feel like crying. | 0.7556521893 |
| STAI-S | I feel tense. | ADS | I have a feeling of dread | 0.7555605173 |
| NEO-FFI (long version) | Often, I don't like the people I have to associate with. | PSS | Sometimes I speak badly about other people. | 0.7553636432 |
| PSS | I'm always in a good mood. | TPF | My mood is good. | 0.7552056909 |
| CBQ | Looks forward to things with excitement. | STAI-S | I am excited. | 0.7548598647 |
| NEO-FFI (long version) | Sometimes I can't assert myself properly. | TPF | There are times when I can't stand myself. | 0.7545668483 |
| CBQ | Cries even with minor injuries. | FBB-DES | Cries a lot. | 0.7545351982 |
| FBB-DES | Appears to lack self-confidence or self-esteem. Appears to feel inferior or like a failure. | STAI-T | I lack self-confidence. | 0.7545249462 |
| EBSK | Things in my life have usually gone against me. | TPF | I am preoccupied with the thought that I have done many things wrong in life. | 0.7543919086 |
| EBSK | I often feel very unsettled. | NEO-FFI (long version) | I often feel tense and nervous. | 0.7543349862 |
| EBSK | I often feel very unsettled. | NEO-FFI (short version) | I often feel tense and nervous. | 0.7543349862 |
| ADHS-LE | I get angry too quickly. | PSS | Others often provoke me until I get angry. | 0.7540184259 |
| SVF | I feel guilty | NEO-FFI (long version) | Sometimes I feel a deep sense of guilt and sin | 0.7539012432 |
| FBB-DES | Has an excessive appetite most of the time. | PSS | I often have a poor appetite. | 0.7538929582 |
| ADHS-LE | I get angry too quickly. | NEO-FFI (long version) | It takes a lot to get me upset. | 0.7538622618 |
| PSS | I believe that people talk a lot about me. | TPF | I am convinced that people can like me a lot. | 0.753651619 |
| EBSK | Too much is expected of me. | TPF | I feel like everything is too much for me. | 0.7532542348 |
| NEO-FFI (long version) | Even minor annoyances can frustrate me. | Social support questionnaire | I often have the desire to annoy others or argue with them. | 0.7524856329 |
| SVF | I can't think of anything else for a long time | TPF | My life is so filled with interesting things that I don't have time to think about myself for long. | 0.7523468733 |
| PSS | I enjoy mocking or ridiculing others. | Social support questionnaire | I really enjoy playing pranks on others. | 0.7523131371 |
| EPDS | I was so unhappy that I had to cry. | STAI-S | I am sad. | 0.7520922422 |
| NEO-FFI (long version) | If I've been hurt, I try to forgive and forget. | TPF | It's difficult for me to forgive the weaknesses and mistakes of my fellow human beings. | 0.7520602942 |
| EBSK | I often feel very alone. | Social support questionnaire | I only feel comfortable when I can be alone. | 0.7519388199 |
| NEO-FFI (long version) | Sometimes I pester people or flatter them to get them to do what I want. | Social support questionnaire | I get annoyed when people don't do what I want. | 0.7516146898 |
| NEO-FFI (long version) | Sometimes frightening thoughts come to my mind. | EPDS | Occasionally, the thought of harming myself crossed my mind. | 0.7513144016 |
| STAI-T | I become nervous and restless when I think about my current affairs. | ADS | I feel more nervous and anxious than usual | 0.7511616349 |
| NEO-FFI (long version) | When I have been alone for a long time, I have a strong need to be with other people. | Social support questionnaire | I prefer to be alone. | 0.7510515451 |
| ADHS-LE | Sometimes I feel helpless against my feelings. | TPF | I have a feeling of indifference and inner emptiness. | 0.7510319948 |
| NEO-FFI (long version) | I am a very active person. | NEO-FFI (short version) | I am a very active person. | 1 |
| NEO-FFI (long version) | I am inspired by the motifs I find in art and nature. | NEO-FFI (short version) | I am inspired by the motifs I find in art and nature. | 1 |
| NEO-FFI (long version) | I enjoy being in the center of attention. | NEO-FFI (short version) | I enjoy being in the center of attention. | 1 |
| NEO-FFI (long version) | I find philosophical discussions boring. | NEO-FFI (short version) | I find philosophical discussions boring. | 1 |
| NEO-FFI (long version) | I have little interest in speculating about the nature of the universe or the state of humanity. | NEO-FFI (short version) | I have little interest in speculating about the nature of the universe or the state of humanity. | 1 |
| NEO-FFI (long version) | I like to have many people around me. | NEO-FFI (short version) | I like to have many people around me. | 1 |
| NEO-FFI (long version) | I often enjoy playing with theories or abstract ideas. | NEO-FFI (short version) | I often enjoy playing with theories or abstract ideas. | 1 |
| NEO-FFI (long version) | I often feel inferior to others. | NEO-FFI (short version) | I often feel inferior to others. | 1 |
| NEO-FFI (long version) | Some people consider me cold and calculating. | NEO-FFI (short version) | Some people consider me cold and calculating. | 1 |
| NEO-FFI (long version) | Sometimes I feel completely worthless. | NEO-FFI (short version) | Sometimes I feel completely worthless. | 1 |
| NEO-FFI (long version) | To get what I want, I am willing to manipulate people if necessary. | NEO-FFI (short version) | To get what I want, I am willing to manipulate people if necessary. | 1 |
| NEO-FFI (long version) | When I commit to something, you can definitely rely on me. | NEO-FFI (short version) | When I commit to something, you can definitely rely on me. | 1 |
| STAI-S | I feel secure. | STAI-T | I feel secure. | 1 |
| STAI-S | I feel rested. | STAI-T | I feel rested. | 1 |
| STAI-S | I am content. | STAI-T | I am content. | 1 |
| STAI-S | I am happy. | STAI-T | I am happy. | 1 |
| STAI-S | I am cheerful. | STAI-T | I am cheerful. | 1 |

**Table S3:** Overview of questionnaire coverage (absolute and relative) generated by Semantic Search Helper during exploration step.

| **Questionnaire** | **Number Of Questions** | **Number Of Item Matches** | **Semantic Coverage (%)** |
| --- | --- | --- | --- |
| NEO-FFI (long version) | 241 | 62 | 25.73 |
| PSS | 134 | 31 | 23.13 |
| TPF | 129 | 30 | 23.26 |
| SVF | 114 | 17 | 14.91 |
| CBQ | 94 | 8 | 8.51 |
| APQ-2 | 72 | 1 | 1.39 |
| Social support questionnaire | 69 | 21 | 30.43 |
| ADHS-LE | 64 | 19 | 29.69 |
| EBSK | 63 | 23 | 36.51 |
| LES | 54 | 5 | 9.26 |
| FBB-ANZ | 33 | 2 | 6.06 |
| KiGa | 30 | 1 | 3.33 |
| NEO-FFI (short version) | 30 | 30 | 100 |
| FBB-DES | 28 | 9 | 32.14 |
| SDQ | 25 | 5 | 20 |
| FBB-SSV | 25 | 5 | 20 |
| IDS | 24 | 4 | 16.67 |
| BDI | 22 | 8 | 36.36 |
| AID | 20 | 6 | 30 |
| STAI-T | 20 | 14 | 70 |
| STAI-S | 20 | 17 | 85 |
| FBB-ADHS | 20 | 4 | 20 |
| ADS | 20 | 9 | 45 |
| ESI | 20 | 3 | 15 |
| EPDS | 10 | 4 | 40 |
| AUDIT | 10 | 4 | 40 |
| Smoking habit interview | 9 | 2 | 22.22 |
| Alcohol consumption Interview | 7 | 1 | 14.29 |
| FTND | 7 | 1 | 14.29 |
